# Polymorphism in microRNA-binding site in *HNF1B* influences the susceptibility of type 2 diabetes mellitus: a population based case–control study

**DOI:** 10.1186/s12881-015-0219-5

**Published:** 2015-09-02

**Authors:** Naoki Goda, Haruna Murase, Nobuhiko Kasezawa, Toshinao Goda, Kimiko Yamakawa-Kobayashi

**Affiliations:** Laboratory of Human Genetics, School of Food and Nutritional Sciences, Graduate School of Integrated Pharmaceutical and Nutritional Sciences, University of Shizuoka, Shizuoka, 422-8526 Japan; Laboratory of Nutritional Physiology, School of Food and Nutritional Sciences, Graduate School of Integrated Pharmaceutical and Nutritional Sciences, University of Shizuoka, Shizuoka, 422-8526 Japan; Department of Data Managements for Health Evaluation & Promotion, Shizuoka Medical Center, Shizuoka, 422-8033 Japan; Present address: Fuji iki-iki Hospital, Health Promotion Center, Temma, Fuji-shi, Shizuoka 419-0205 Japan

## Abstract

**Background:**

Recent genome-wide association studies (GWAS) have identified many SNPs associated with type 2 diabetes mellitus (T2DM). However, the functional roles for most of the SNPs have not been elucidated. MicroRNAs (miRNAs) are key regulators of gene expression involved in the development and progression of various diseases including T2DM. In this study, we investigated whether commonly occurring SNPs modulate miRNA-directed regulation of gene expression, and whether such SNPs in miRNA-binding sites are associated with the susceptibility for T2DM.

**Methods:**

Genotypes of eleven 3′ untranslated region (UTR) SNPs of seven susceptibility genes for T2DM were determined in 353 T2DM patients and 448 control subjects. In addition, the interactions of miRNAs with the 3′UTR in the hepatocyte nuclear factor 1β (*HNF1B*) gene were investigated using luciferase reporter assays.

**Results:**

One 3′UTR SNP (rs2229295) in the *HNF1B* gene was significantly associated with T2DM, and the frequency of an A allele (rs2229295) in T2DM patients was decreased compared with that in controls. Luciferase reporter assays showed that the SNP (rs2229295) altered the binding of two miRNAs (hsa-miR-214-5p and hsa-miR-550a-5p).

**Conclusions:**

We have detected the interactions of hsa-miR-214-5p/hsa-miR-550a-5p and the 3′UTR SNP of the *HNF1B* gene by *in vitro* luciferase reporter assays, and propose that the binding of such miRNAs regulates the expression of the *HNF1B* gene and the susceptibility of T2DM.

**Electronic supplementary material:**

The online version of this article (doi:10.1186/s12881-015-0219-5) contains supplementary material, which is available to authorized users.

## Background

Type 2 diabetes mellitus (T2DM) is a common heterogeneous and complex disease that is characterized by hyperglycemia resulting from impaired pancreatic β-cell function and a decreased action of insulin on target tissues. A combination of multiple genetic and environmental factors is considered to contribute to the pathogenesis of this disease. Patients with T2DM are at greater risk of developing cardiovascular diseases, renal failure, neurological conditions, and retinopathy [1–3] Recent genome-wide association studies (GWAS) have successfully identified over 65 susceptibility loci associated with T2DM and related metabolic traits [4–6]. GWAS have been a powerful approach to identify single nucleotide polymorphisms (SNPs) associated with disease risk. However, most of the SNPs in susceptibility genes for T2DM identified in previous studies were located within non-translated regions, such as introns, 3′-untranslated regions (3′UTRs), and 5′UTRs. Therefore, functional roles for many of the SNPs in susceptibility genes have not been elucidated.

MicroRNAs (miRNAs) are endogenous noncoding RNAs (19–25 nucleotides in length) that induce the translational repression and degradation of target mRNAs by complementarily binding to their 3′UTR [7]. By silencing their target gene expression, miRNAs are involved in a variety of biological processes, as well as the development and progression of human diseases including cancer and T2DM [8–13]. Previous studies showed that SNPs within or proximal to miRNA-binding sites in target genes have the potential to either create or destroy binding sites, which affects the efficiency of miRNA binding on target sites. Thus, SNPs in miRNA-binding sites may modulate expression and protein levels of target genes, and ultimately contribute to phenotypic variations, including disease susceptibility and important traits [9–11, 14].

In this study, we investigated whether commonly occurring SNPs modulate miRNA-directed regulation of gene expression, and whether such SNPs in miRNA-binding sites are associated with the susceptibility for T2DM.

## Methods

### Subjects

The participants recruited for this study were Japanese who underwent a routine medical check-up at a medical center near the University of Shizuoka. We selected men under 65 years of age as subjects in this study. The case subjects with T2DM (*n* = 353) were diagnosed as T2DM by physicians according to the World Health Organization (WHO) diagnostic criteria for T2DM [15]. Of these, 251 T2DM patients (71.1 %) were under oral medication for diabetes. The control subjects (*n* = 448) were randomly selected according to the following criteria to exclude persons with potential glucose intolerance: (1) fasting plasma glucose levels were under 100 mg/dL (5.6 mmol/L), and (2) HbA1c levels were under 6.2 %. All subjects provided written informed consent to participate in this study, and the study was approved by the Ethics Committee of the University of Shizuoka (Approval No. 17-1)

After overnight fasting, blood was collected from each subject. The clinical characteristics of the subjects were determined according to the medical check-up protocol (Table 1).

**Table 1:** Characteristics of the study subjects

	T2DM	Control	*P* -value
	*n* = 353	*n* = 448	
Age (years)	54.4 ± 6.5	53.7 ± 5.1	0.12
BMI (kg/m^2^)	25.0 ± 3.6	23.1 ± 2.6	<0.0001
Glucose (mg/dl)^*^	155.0 ± 43.2	91.8 ± 4.9	<0.0001
HbA1c (%)	7.7 ± 1.57	5.4 ± 0.34	<0.0001
SBP (mmHg)	126.3 ± 15.6	120.5 ± 16.2	<0.0001
DBP (mmHg)	79.9 ± 11.0	76.8 ± 12.0	0.0002
Total-cholesterol (mg/dl)	212.5 ± 34.7	210.2 ± 31.7	0.33
LDL-cholesterol (mg/dl)	130.8 ± 29.9	129.5 ± 29.9	0.55
HDL-cholesterol (mg/dl)	54.0 ± 15.9	58.4 ± 16.4	0.0001
Triglyceride (mg/dl)^*^	161.8 ± 145.0	136.3 ± 112.8	0.0001
Obesity/Overweight (BMI ≥ 25) (%)	45.0	23.2	<0.0001
Current smoker (%)	43.7	41.8	0.61

^*^Statistical test for glucose and triglyceride levels were caluculated on log-transformed values

*P*-values between T2DM and control groups were caluculated by *t*-test or χ^2^ -test

Data are expressed as mean ± SD or percentage

### DNA analysis

We first selected 20 risk genes for T2DM, which contains 17 risk genes that had been examined the association for T2DM in our previous study [16], and three risk genes *(PPARGC1A, IRS2,* and *SPRY2)* [17–19]. These 20 genes were confirmed the association with T2DM in Asian populations [4, 5]. Next, we searched SNPs in the miRNA-binding sites in the 3′UTR of these 20 T2DM risk genes using three online databases: MirSNP [20, 21], PolymiRTS database 3.0 [22, 23], and miRNASNP [24, 25]. Finally, we selected 11 SNPs with minor allele frequency >0.05 in Japanese in the 3′UTR of seven T2DM susceptible genes (Table 2).

**Table 2:** Associations between T2DM and 3′ UTR SNPs of susceptibility genes for T2DM

Gene	SNP	Predicted interacting miRNA	Genotype	T2DM n (%)	Control n (%)	OR (95%CI)	*P*-value	Power
*SLC30A8*	rs11558471	hsa-miR-1205	AA	130 (37.3)	135 (30.7)	1 (Reference)	0.022	0.78
		hsa-miR-1248	AG	168 (48.1)	229 (52.1)	0.78 (0.62–0.96)		
		hsa-miR-3074-5p	GG	51 (14.6)	76(17.3)	0.60 (0.39–0.93)		
	rs3802178	hsa-miR-1234-3p	AA	145 (41.6)	215 (48.4)	1 (Reference)	0.10	0.74
			GA	169 (48.4)	187 (42.1)	1.21 (0.96–1.51)		
			GG	35 (10.0)	42 (9.5)	1.46 (0.93–2.30)		
	rs2466295	hsa-miR -586	TT	275 (77.9)	341 (76.8)	1 (Reference)	0.67	0.52
			TC	71 (20.1)	91 (20.5)	0.94 (0.69–1.27)		
			CC	7 (2.0)	12 (2.7)	0.88 (0.48–1.60		
	rs2466293	hsa-miR- 181a-2-3p	TT	95 (27.3)	145 (33.2)	1 (Reference)	0.11	0.78
		hsa-miR-660-5p	TC	181 (52.0)	203 (46.5)	1.19 (0.96–1.47)		
		hsa-miR-888-3p	CC	72 (20.7)	89 (20.4)	1.41 (0.93–2.15)		
		hsa-miR-1273d						
*HNF1B*	rs2229295	hsa-miR214-5p	CC	278 (78.7)	308 (68.8)	1 (Reference)	**0.004**	0.61
		hsa-miR550a-5p	CA	66 (18.7)	121 (27)	0.66 (0.50–0.88)		
		hsa-miR550a-3-5p	AA	9 (2.6)	19 (4.2)	0.44 (0.25–0.77)		
		hsa-miR1271-3p						
	rs1800929	hsa-miR214-5p	AA	238 (67.4)	285 (63.6)	1 (Reference)	0.071	0.65
		hsa-miR550a-5p	AG	100 (28.3)	142 (31.7)	0.79 (0.60–1.02)		
		hsa-miR550a-3-5p	GG	15 (4.3)	21 (4.7)	0.62 (0.37–1.04)		
		hsa-miR1271-3p						
*CDC123*	rs10951	hsa-miR-145-5p	GG	169 (53.5)	249 (60.9)	1 (Reference)	0.027	0.65
		hsa-miR-770-5p	GA	134 (42.4)	147 (35.9)	1.36 (1.04–1.79)		
		hsa-miR-4712-5p	AA	13 (4.1)	13 (3.2)	1.86 (1.07–3.22)		
*PPARGC1A*	rs6821591	hsa-miR-187-3p	TT	180 (55.7)	200 (48.7)	1 (Reference)	0.13	0.73
		hsa-miR-595	TC	120 (37.2)	180 (43.8)	0.83 (0.65–1.06)		
			CC	23 (7.1)	31 (7.5)	0.69 (0.42–1.12)		
*UBE2E2*	rs7631705	hsa-miR-888-3p	TT	155 (47.8)	165 (42.5)	1 (Reference)	0.018	0.76
		hsa-miR-3660	TC	143 (44.1)	170 (43.8)	0.75 (0.59–0.95)		
		hsa-miR-4526	CC	26 (8.0)	53 (13.7)	0.57 (0.35–0.91)		
*IRS2*	rs2289047	hsa-miR-376c-3p	GG	103 (29.6)	141 (32.3)	1 (Reference)	0.22	0.78
			GT	163 (46.8)	215 (49.3)	1.14 (0.92–1.40)		
			TT	82 (23.6)	80 (18.4)	1.29 (0.85–1.96)		
*SPRY2*	rs1644394	hsa-miR-1224-3p	TT	199 (57.0)	255 (58.5)	1 (Reference)	0.77	0.69
		hsa-miR-1260a	TG	124 (35.5)	146 (33.5)	0.97 (0.77–1.22)		
		hsa-miR-1260b	GG	26 (7.5)	35 (8.0)	0.93 (0.59–1.48)		
		hsa-miR-4733-5p						

Odd ratios and *P* - values were adjusted by age and BMI

Statistically significant *P* - value after Bonferroni correction was indicated in bold

Power to detect association was estimated under current sample size and minor allele frequency, assuming OR = 1.2 and additive effect

Genomic DNA was isolated from peripheral leukocytes by the phenol extraction method. The genotypes of the SNPs were determined for each subject using the PCR-restriction fragment length polymorphism method.

### *HNF1B* 3′UTR reporter gene construction

Two SNPs (rs2229295 C > A, rs1800929 A > G) lie next to each other in the microRNA binding sites in the 3′UTR of the hepatocyte nuclear factor 1B *(HNF1B)* gene. The *HNF1B* 3′UTR (920 bp) was amplified using PrimeSTAR® HS DNA Polymerase (Takara Bio Inc., Otsu, Japan) from the genomic DNA of the homozygote for major alleles of the two SNPs (C for rs2229295, A for rs1800929). The primer sequences are listed in Additional file 1.

The purified PCR product was subcloned into pUC18 vector. We then generated five distinct reporter gene constructs containing sequences as follows: (1) C (rs2229295), A (rs1800929), (2) C (rs2229295), G (rs1800929), (3) A (rs2229295), A (rs1800929), (4) A (rs2229295), G (rs1800929), and (5) T, C (as a reference; randomly selected nucleotide sequence), by site-directed mutagenesis using PrimeSTAR® Mutagenesis Basal Kit (Takara Bio). The primer sequences used in the site-directed mutagenesis are listed in Additional file 1. These inserts were removed by digestion with *Sma* I and *Hind* III, and cloned downstream of the luciferase gene in a reporter vector; pMIR-REPORT™ Luciferase (Ambion Inc., Austin, TX, USA). Each construct was sequenced to confirm the sequence and orientation of the insert.

### Luciferase reporter assay

HEK293 cells (a human embryonic kidney cell line) were cultured in Dulbecco’s Modified Eagle’s medium with 10 % fetal bovine serum. The cells were seeded in 24-well plates 24 h before transfection. When the cells were grown to about 80–90 % confluence, the reporter plasmid (150 ng/well) and miRNA mimics (5 pmol/well) (Bioneer Inc., Daejeon, Korea) were transfected using Lipofectamine 2000 (Invitrogen, Inc., Carlsbad, CA, USA) according to the manufacturer’s instruction. The phRG-TK vector containing *Renilla reniformis* luciferase (6.5 ng/well) (Promega Inc., Madison, WI, USA) was also co-transfected to standardize transfection efficiency. After 24 h, luciferase activity in cell lysate was measured using the Dual-Luciferase Reporter Assay System (Promega). Three independent transfection experiments were performed in triplicate.

### Statistical analyses

The associations of genotypes of the eleven 3′ UTR SNPs in seven T2DM susceptibility genes and T2DM were examined. The genotype specific odds ratios (ORs) with 95 % confidence intervals (CIs) and *p*-values for T2DM were calculated using logistic regression analysis, adjusting for age and BMI.

In the luciferase reporter assay, the differences in the luciferase activity between four kinds of constructs (CA, CG, AA and AG) were examined by Tukey-Kramer multiple comparisons test. All statistical analyses were performed using the JMP software package (SAS Institute, Cary, NC, USA). The power to detect an association between each SNP and T2DM was estimated under current sample size and minor allele frequency observed in this study using “Quanto” [26], assuming OR = 1.2, α level = 0.05 (one-sided), and additive model.

For association between T2DM and each SNP, *p* < 0.0045 (0.05/11) was considered as significant by applying a Bonferroni correction.

## Results

We analyzed the relationships between T2DM and genotypes of eleven 3′UTR SNPs in seven T2DM susceptibility genes that were previously detected by GWAS. The genotype distributions of these 11 SNPs were in Hardy-Weinberg equilibrium (*P* > 0.05). Table 2 shows the associations between T2DM and these SNPs. The ORs and *p*-values were adjusted for age and BMI in logistic regression analysis. One 3′UTR SNP (rs2229295) in the *HNF1B* gene was significantly associated with T2DM, and the frequency of CA and AA genotypes of rs2229295 in T2DM patients was decreased compared with that in controls (OR = 0.66 (95 % CI: 0.50–0.88), 0.44 (95 % CI: 0.25–0.77), respectively)) (Table 2). These data indicate that the A allele of 3′UTR SNP (rs2229295) in the *HNF1B* gene can be a protective allele for T2DM. The other ten 3′UTR SNPs in the susceptibility genes were not associated with T2DM.

**Fig. 1:**
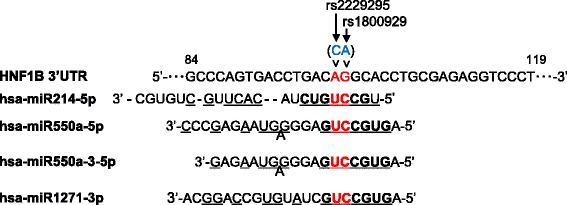
Predicted miRNAs whose binding are possibly affected by the base substitutions due to SNPs r22229295 and rs1800929. The four miRNAs were predicted as candidate miRNAs in at least two of three online databases (MirSNP, PolymiRTS, and miRNASNP) [20–25]. Seed sequences of each miRNA were indicated by bold. The complemetary sequences of 3′UTR of the *HNF1B* gene were shown by underlined. The red color showed sites for SNPs (rs2229295 and rs1800929)

To investigate the functional impact of the SNP (rs2229295) in the *HNF1B* gene, we next searched miRNAs whose binding could be affected by the base substitution due to this SNP (rs2229295) using online databases (MirSNP, PolymiRTS, and miRNASNP). We identified four candidate miRNAs whose seed sequences correspond with complementary sequences around the SNP (rs2229295) (Fig. 1). In this region, two SNPs (rs2229295 C > A, rs1800929 A > G) are located next to each other. In addition, the seed sequences of these four miRNA contain complementary sequences to the minor alleles of two SNPs (A for rs2229295, G for rs1800929) of the *HNF1B* gene (Fig. 1).

**Fig. 2:**
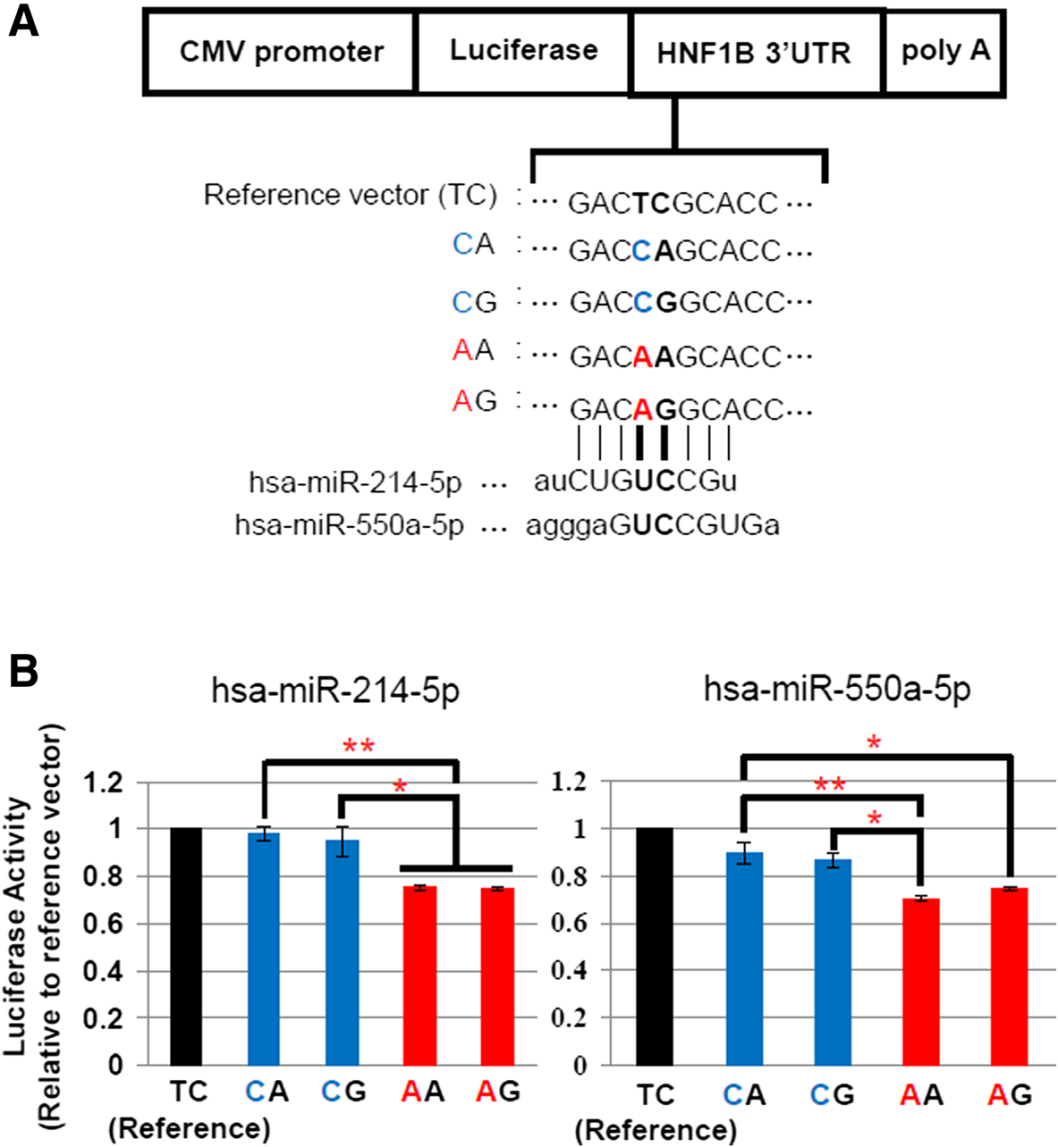
Effect of the base substitutions due to SNPs rs2229295 and rs1800929 on miRNA binding. **a** Schematic representation of reporter constructs used in the luciferase reporter assay. Plasmid construct containing TC sequence, which was selected randomly, was used as a reference. Major allele (C for rs2229295) is shown in blue and minor allele (A for rs2229295) is shown in red. **b** Relative luciferase activity of each reporter construct. Luciferase activity was normalized to *Renilla* luciferase levels. Luciferase activities relative to the reference vector (TC vector) are shown as mean ± S.E. from three independent transfection experiments with triplicate assays. The luciferase activities among four constructs were compared using the Turkey-Kramer method (*p < 0.05, **p < 0.01)

Next, we tested whether the binding of these four miRNAs to the 3′UTR of the *HNF1B* gene was affected by the two SNPs. We generated four kinds of luciferase reporter constructs and one reference construct as described in Methods (Fig. 2a). The constructs were each co-transfected in parallel with the four predicted candidate miRNA mimics into HEK293 cells, and luciferase activity was compared. When hsa-miR-214-5p or hsa-miR-550a-5p mimics were co-transfected with the reporter construct, significant suppression of luciferase activity was observed in constructs containing AA or AG sequences for the two SNPs (rs2229295, rs1800929) compared with the construct containing CA sequence, which presumably does not bind miRNAs (Fig. 2b). When the other two miRNA mimics (hsa-miR-550a-3-5p, hsa-miR-1271-3p) were co-transfected with each reporter construct, there were no differences in luciferase activity among reporter construct (Additional file 2). Furthermore, there were no differences in luciferase activities among reporter constructs when they were transfected into HEK293 cells without miRNA mimics (Additional file 3).

These data indicate that the substitution of C > A due to SNP (rs2229295) induces a decrease of luciferase activity. However, A > G substitution due to SNP (rs1800929) did not affect luciferase activity. The results of luciferase reporter assays showed that the SNP (rs2229295) actually alters the binding of two miRNAs (hsa-miR-214-5p and hsa-miR-550a-5p), and A allele carrying constructs were specifically regulated by the two miRNAs, while the adjacent SNP (rs1800929) did not affect the binding of the miRNAs to *HNF1B* 3′UTR.

## Discussion

Previous studies have demonstrated that genetic variations within miRNA-binding sites could modulate gene expression and protein levels, and affect phenotypes or cause disease [8–10]. In this study, we identified an SNP (rs2229295) in the 3′UTR of the *HNF1B* gene that could affect miRNA binding and that was associated with the risk of T2DM. Two SNPs (rs2229295, rs1800929) lie next to each other in this region. *In silico* analysis predicted that substitutions C > A in rs2229295 and A > G in rs1800929 create a new potential miRNA-binding site in the 3′UTR of the *HNF1B* gene (Fig. 1). It was ascertained one SNP (C > A in rs2229295) could affect the binding of two miRNAs (hsa-miR-214-5p, hsa-miR-550a-5p) by luciferase reporter assay. However, the other SNP (rs1800929) and two miRNAs (hsa-miR-550a-3-5p, hsa-miR-1271-3p) did not influence the luciferase activity. Many potential miRNA target sites can be predicted in 3′UTRs of many genes by *in silico* analysis. However, the binding of miRNAs and target genes have considerable flexibility and therefore *in silico* analysis is not sufficient to define 3′UTR SNPs related to susceptibility of common diseases.

HNF1B is a homeodomain-containing transcription factor expressed in multiple tissues, such as the liver, kidney, pancreas, and genital tract [27, 28]. Mouse studies revealed that HNF1B is a critical regulator of transcriptional network that controls the specification, growth, and differentiation of the embryonic pancreas [29–31]. In humans, mutations in the *HNF1B* gene were described in a monogenic form of diabetes, namely maturity-onset diabetes of the young type 5 (MODY5) [28, 32–35]. Many patients with MODY5 have impaired insulin secretory responses to glucose and show progressive loss in basal insulin secretion, although they have various other abnormalities such as renal cysts, renal impairment, and genital malformation [28–35].

Some GWAS revealed that several tag SNPs in the *HNF1B* gene were associated with the susceptibility of T2DM, and such associations were well replicated in many countries [36–38]. However, the SNP (rs 2229295) that was associated with the risk of T2DM in this study was not a tag SNP for the *HNF1B* gene. There is no report for the association of this SNP (rs 2229295) and T2DM. We could not observe significant linkage disequilibrium (LD) between the SNP (rs 2229295) and a tag SNP (rs7501939) of the *HNF1B* gene (Additional file 4).

Recently, Kornfeld and colleagues found that obesity-induced overexpression of miR-802 causes glucose intolerance, impairs insulin signaling, and promotes hepatic gluconeogenesis in the liver through direct silencing of *HNF1B,* and showed an important role for HNF1B in the control of hepatic insulin sensitivity and glucose metabolism *in vivo* [39].

We have detected the interactions of hsa-miR-214-5p/hsa-miR-550a-5p and the 3′UTR of the *HNF1B* gene by *in vitro* luciferase reporter assays, and our results suggest that binding of hsa-miR-214-5p and hsa-miR-550a-5p may also regulate the expression of the *HNF1B* gene. Unfortunately, we could not examine the interactions between such miRNAs and the endogenous *HNF1B* gene. Because the genomic sequence of miRNA binding site of the *HNF1B* gene in HEK293 cells that we used in this study is C (rs2229295), this sequence does not bind hsa-miR-214-5p and hsa-miR-550a-5p. Furthermore, we have no data as to whether HNF1B mRNA and/or protein levels *in vivo* are affected by the genotype of the SNP (rs2229295).

The *miR214* gene is located in an intronic region of the *Dynamin-3* gene on human chromosome 1q24.3, and is expressed in the liver, kidney, pancreas, and osteoblasts involved in the development of pancreas and bone [40, 41]. The *miR-550* gene is located in the intronic region of the *Znrf2* gene on human chromosome 7p14.3, and expressed in multiple cancers including hepatocellular carcinoma [42]. However, there is little information regarding the function and regulation of expression of *miR-550* in normal cells and tissues. We need to know how the expressions of hsa-miR-214-5p and hsa-miR-550a-5p are regulated *in vivo.*

In this study, we found the possibility that the binding of two miRNAs to the 3′UTR of the *HNF1B* gene provided the protective effect for T2DM. In most patients with MODY5, the clinical phenotypes may be related to loss of function or dominant-negative mechanisms for HNF1B [28, 32–35]. However, a previous study reported a mutation that showed a gain-of function phenotype with increased transcript activity of the *HNF1B* gene [43]. Important roles of HNF1B for complex transcriptional networks in pancreatic β-cells and hepatocytes have been established [35, 44, 45]. There is a possibility that the dysregulated expression of the *HNF1B* gene due to nucleotide changes within the miRNA-binding site would lead to impair transcriptional networks related to HNF1B and the differences of susceptibility for T2DM. Further experiments are needed to ascertain roles for hsa-miR-214-5p and hsa-miR-550a-5p and HNF1B-dependent regulation of insulin secretion, glucose metabolism *in vivo.*

## Conclusions

In this study, we found the 3′UTR SNP (rs2229295) in the *HNF1B* gene was associated with the susceptibility of T2DM. In addition, luciferase reporter assays indicate that the substitution of C > A due to SNP (rs2229295) induces the binding of hsa-miR-214-5p/hsa-miR-550a-5p to the 3′UTR of the *HNF1B* gene.

There is a possibility that the dysregulated expression of the *HNF1B* gene due to nucleotide changes within miRNA binding site lead the difference of susceptibility for T2DM.

## Additional files

Additional file 1: Table S1. PCR primers used for subcloning and introduction of nucleotide changes in 3′UTR of *HNF1B*. (XLSX 11 kb) Additional file 2: Figure S1. Effect of miRNA (A:hsa-miR-550a-3-5p, B: hsa-miR1271-3p) binding to reporter constructs. There was no significant difference in luciferase activities among constructs containing CA, AA, or AG sequences (for SNP rs2229295 and rs1800929). Luciferase activities relative to reference vector (TC vector) were shown as mean ± S.E. from 3 independent transfection experiments with triplicate assays. The comparisons of luciferase activity among four constructs were using Turkey-Kramer method. (PDF 10 kb) Additional file 3: Figure S2. Effect of the base substitutions due to SNPs (rs2229295 and rs1800929) to luciferase activity. There was no significant difference in luciferase activity of each reporter constract containing CA, CG, AA, AG sequence, suggesting that the difference in 3′UTR sequence due to SNPs (rs2229295 and rs1800929) did not affect the luciferase activity by itself. Luciferase activity was normalized to *Renilla* luciferase levels. Luciferase activities relative to reference vector (TC vector) were shown as mean ± S.E. from 3 independent transfection experiments with triplicate assays. The comparison of luciferase activity among four constructs were using Turkey-Kramer method. (PDF 7 kb) Additional file 4: Table S2. The pairwise linkage disequilibrium (LD) values of |D'| (upper) and r2 (lower) (XLSX 11 kb)

## Abbreviations

GWAS: Genome-wide association studies
SNP: Single nucleotide polymorphism
T2DM: Type 2 diabetes mellitus, miRNA: microRNA
3′ UTR: 3′ Untranslated region
HNF1B: Hepatocyte nuclear factor 1 beta
WHO: World Health Organization
HbA1c: Hemoglobin A1c
PCR: Polymerase chain reaction
ORs: Odds ratios
CI: Confidence intervals
SLC30A8: Solute carrier family 30 (zinc transporter), member 8
CDC123: Cell division cycle 123
PPARGC1A: Peroxisome proliferator-activated receptor gamma, coactivator 1 alpha
UBE2E2: Ubiquitin-conjugating enzyme E2E 2
IRS2: Insulin receptor substrate 2
SPRY2: Sprouyty drosophila homolog of 2
