# Supplementary figures and images for: Polymorphism in microRNA-binding site in HNF1B influences the susceptibility of type 2 diabetes mellitus: a population based case–control study
Source: BMC Med Genet. 2015 Sep 2;16:75. doi: 10.1186/s12881-015-0219-5 (PMC4557749; doi:10.1186/s12881-015-0219-5)

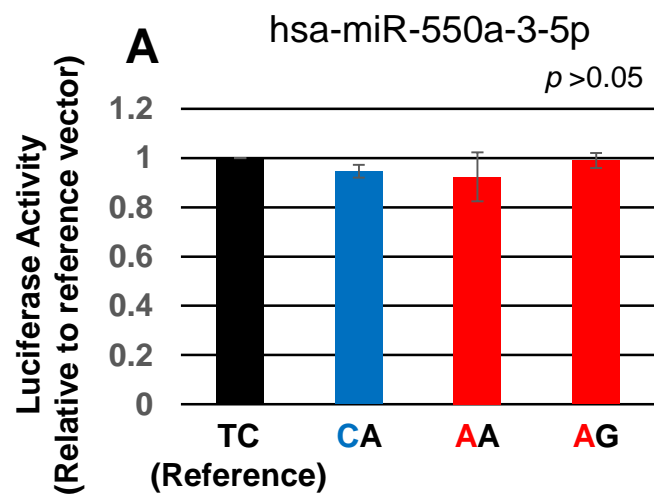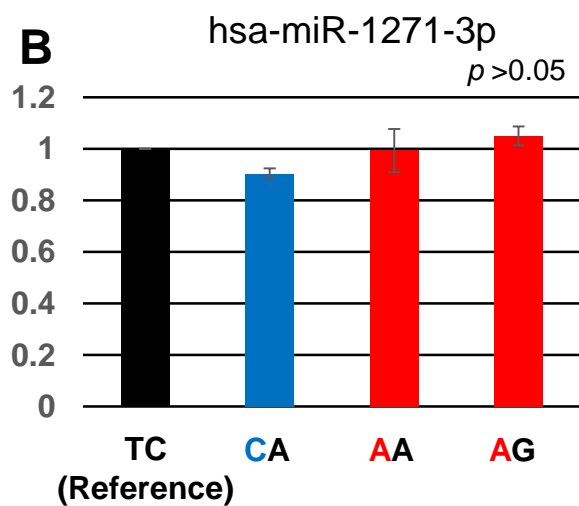

Supplement: Additional file 2: Figure S1. — Effect of miRNA (A:hsa-miR-550a-3-5p, B: hsa-miR1271-3p) binding to reporter constructs. There was no significant difference in luciferase activities among constructs containing CA, AA, or AG sequences (for SNP rs2229295 and rs1800929). Luciferase activities relative to reference vector (TC vector) were shown as mean ± S.E. from 3 independent transfection experiments with triplicate assays. The comparisons of luciferase activity among four constructs were using Turkey-Kramer method. (PDF 10 kb) [file 12881_2015_219_MOESM2_ESM.pdf]

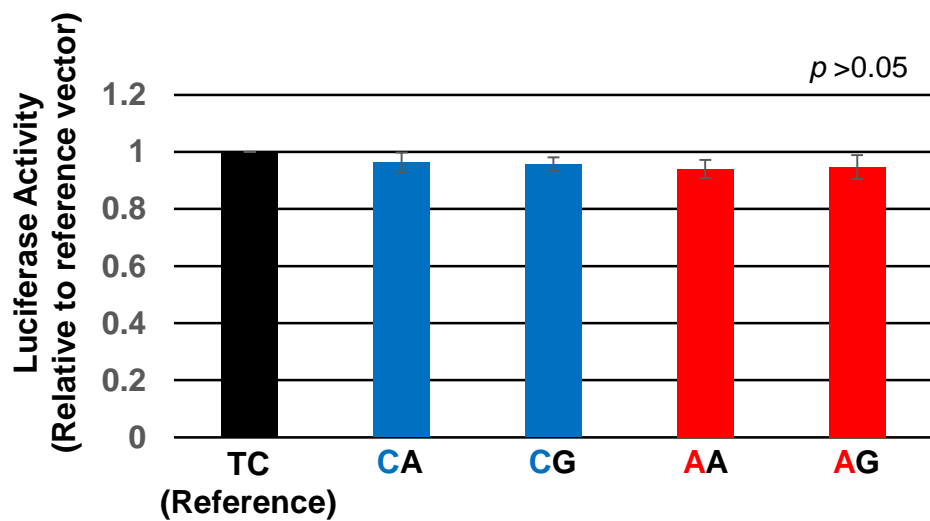

Supplement: Additional file 3: Figure S2. — Effect of the base substitutions due to SNPs (rs2229295 and rs1800929) to luciferase activity. There was no significant difference in luciferase activity of each reporter constract containing CA, CG, AA, AG sequence, suggesting that the difference in 3′UTR sequence due to SNPs (rs2229295 and rs1800929) did not affect the luciferase activity by itself. Luciferase activity was normalized to Renilla luciferase levels. Luciferase activities relative to reference vector (TC vector) were shown as mean ± S.E. from 3 independent transfection experiments with triplicate assays. The comparison of luciferase activity among four constructs were using Turkey-Kramer method. (PDF 7 kb) [file 12881_2015_219_MOESM3_ESM.pdf]
